# ^1^H NMR-based metabolic profiling of urinary tract infection: combining multiple statistical models and clinical data

**DOI:** 10.1007/s11306-012-0411-y

**Published:** 2012-02-29

**Authors:** Ekaterina Nevedomskaya, Tiziana Pacchiarotta, Artem Artemov, Axel Meissner, Cees van Nieuwkoop, Jaap T. van Dissel, Oleg A. Mayboroda, André M. Deelder

**Affiliations:** 1 Biomolecular Mass Spectrometry Unit, Department of Parasitology, Leiden University Medical Center, Albinusdreef 2, 2333ZA Leiden, The Netherlands; 2 Department of Infectious Diseases, Leiden University Medical Center, Leiden, The Netherlands; 3 Present Address: Department of Internal Medicine, Haga Hospital, The Hague, The Netherlands

**Keywords:** Metabolomics, NMR, Data analysis, Clinical metabolomics, Urinary tract infection

## Abstract

Urinary tract infection (UTI) encompasses a variety of clinical syndromes ranging from mild to life-threatening conditions. As such, it represents an interesting model for the development of an analytically based scoring system of disease severity and/or host response. Here we test the feasibility of this concept using ^1^H NMR based metabolomics as the analytical platform. Using an exhaustively clinically characterized cohort and taking advantage of the multi-level study design, which opens possibilities for case–control and longitudinal modeling, we were able to identify molecular discriminators that characterize UTI patients. Among those discriminators a number (e.g. acetate, trimethylamine and others) showed association with the degree of bacterial contamination of urine, whereas others, such as, for instance, scyllo-inositol and para-aminohippuric acid, are more likely to be the markers of morbidity.

**Electronic supplementary material:**

The online version of this article (doi:10.1007/s11306-012-0411-y) contains supplementary material, which is available to authorized users.

## Introduction

Despite the progress made to date in understanding the mechanistic basis of many diseases, medicine is still essentially “more an art than a science” (Woodcock 2007). Specific and sensitive biological markers are important contributors to the improved diagnostic methods as well as to patient care and drug discovery. Advanced “-omics” technologies, such as genomics, proteomics and metabolomics, enable identification of such markers (Vaidya and Bonventre 2010). Of our particular interest is metabolomics that focuses on the analysis of metabolites present in biological fluids. Metabolites are end-points of all the biochemical processes of the organism and thus their collection—the metabolome is the closest approximation of the physiological phenotype and as such has a great potential for uncovering the biology underlying diseases and providing valuable markers of pathology (Lindon and Nicholson 2008; Holmes et al. 2008).

The biological interpretation of results from metabolomics studies is rather complex and still in an early phase of development (Mendes and Camacho 2005). The human body is a “super-organism” that unites its own network of interconnected tissues and organs with multiple colonies of microorganisms (Nicholson et al. 2005). Interpretation of changes in concentration of metabolites found in biological fluids can readily be performed based on the underlying metabolic pathway; however, it is not always possible to link the observed change in systemic metabolite concentrations to a specific tissue or organ (Adourian et al. 2008). Especially in the case of disruption of highly abundant metabolites, e.g. from energy or amino acid metabolism, additional information would be required in order to interpret the data in respect to the tissue of origin. In addition, a change of such metabolites does not always improve the knowledge about the underlying cellular mechanisms and biology. A way to facilitate the interpretation of clinical metabolomics data is to integrate a plethora of available clinical parameters and to utilize a multilevel study design that should provide the opportunity to access the various levels of biological processes.

One of the examples of a complex and heterogeneous clinical entity, for which current diagnostic methods are not straightforward, is urinary tract infection (UTI) (Wilson and Gaido 2004). Clinical manifestations of UTI can cover the range from mild cystitis to advanced pyelonephritis potentially leading to urosepsis and multiple-organ failure. Physical symptoms may vary from patient to patient and be similar to a number of other diseases, mainly of infectious origin. Thus, the presence of bacteria and leucocytes in urine can not be considered as a sole common denominator for UTI and even if it was, the criterion for the colony count is variable and anyway considered insensitive (Johnson 2004). The correct and timely diagnosis relies on effective joint work of clinicians and microbiologists (Johnson 2004). All of this explains the considerable interest in providing new, specific and sensitive markers for UTI and for the uropathogen involved. The focus of the available metabolomics studies on UTI in the literature has so far been on the identification of pathogens: in the work of Gupta et al*.* a beautiful method with the use of ^1^H NMR was proposed (Gupta et al. 2006, 2009; Gupta and Dwivedi 2005). However, the method is targeted solely to the pathogens, leaving all host-related questions, such as localization of the infection within urinary tract and morbidity, unexplored.

In the current study we investigated possibilities of using urinary metabolic profiles to monitor the health state of UTI patients, the degree of infection and the recovery process of UTI patients in the context of febrile, complicated UTI. We used a selection of samples from an exhaustively characterized cohort, with multiple urine samples available per individual and with the main pathogen identified as *Escherichia coli*, which is the most common pathogen for UTI. Samples from a group of age- and gender- matched UTI symptom-free subjects were included as control. The longitudinal design allowed studying various biological processes: not only the difference between the patients and controls, but also the recovery process, using each patient as its own control.

## Materials and methods

### Samples

The study protocol was approved by the ethical committee of the Leiden University Medical Center and all included patients gave written informed consent.

Urine samples were collected at the Emergency Department and Primary Care Department from adult patients presenting with febrile UTI. The methods have been described previously (van der Starre et al. 2011; van Nieuwkoop et al. 2010). The sampling was carried out at several time points: the first urine samples were collected at the day of enrolment as baseline samples (*t* = 0). Clean midstream-catch urine cultures were obtained and were analyzed using local standard microbiological methods. Three-four (*t* = 4) and 30 days (*t* = 30) after the day of enrolment, urine samples of the same patients were collected and new bacterial culture tests were performed (Supplementary Fig. 1).

For the current study, from a database of 253 subjects enrolled, 40 subjects, for which urine culture confirmed *E. coli*-positive complicated febrile UTI infection that recovered after antibiotic treatment, were randomly selected (Supplementary Fig. 1). Samples from age- and gender- matched subjects with low bacterial culture in urine and without evidence of inflammatory diseases were used as controls (Table 1). A number of samples were missing, a few removed from the analysis due to either insufficient spectra quality or high glucose content (Supplementary Fig. 1). In the end the study included four classes of samples originating from UTI symptom-free (*N* = 35) at day 0 (baseline control), UTI patients (*N* = 32) at day 0 (baseline), UTI patients (*N* = 29) at day 4 and UTI patients after recovery from infection (*N* = 37) at day 30 (Supplementary Fig. 1).

**Table 1:** Characteristics of the studied patients and controls groups at baseline (*t* = 0)

Characteristics	UTI patients	Controls	*P* value
	*N* = 40	*N* = 40	
Age, years, median (sd)	59 (14.6)	58 (17.9)	0.9
Female, *N* (%)	22 (55)	22 (55)	1
Smoking, *N* (%)	5 (12)	5 (12)	1
Co-morbidity, *N* (%)			
Urinary tract disorder	4 (10)	4 (10)	1
Malignancy	4 (10)	1 (3)	0.17
Heart failure	5 (13)	3 (8)	0.46
Renal insufficiency	1 (4)	0 (0)	0.13
Diabetes mellitus	6 (15)	2 (5)	0.14
Immunocompromised	1 (3)	1 (3)	1
Urine dipstick results			
Nitrite	26/37 (75)^a^	0/37 (0)^a^	<0.001
Leucocyte esterase	35/37 (95)^a^	5/37 (14)^a^	<0.001

^a^3 missing values

### Sample preparation

Samples were thawed, transferred into 96 deep-well plates and centrifuged at 3,000×*g* for 15 min at 4°C to remove any precipitate. For sample preparation 520 μl urine were mixed with 60 μl of pH 7.0 phosphate buffer (1.5 M) in 100% D_2_O containing 4 mM sodium 3-trimethylsilyl-tetradeuteriopropionate (TSP) and 2 mM NaN_3_ in a 96 deep-well plate using a Gilson 215 liquid handler controlled by a Bruker Sample Track LIMS system (Bruker BioSpin, Karlsruhe, Germany).

### NMR experiments and processing

^1^H NMR data were collected using a Bruker 600 MHz AVANCE II spectrometer equipped with a 5 mm TCI cryogenic probe head and a z-gradient system; a Bruker BEST (Bruker Efficient Sample Transfer) system was used in combination with a 120 μl CryoFIT^™^ flow insert for sample transfer. One-dimensional (1D) ^1^H NMR spectra were recorded at 300 K using the first increment of a NOESY pulse sequence (Kumar et al. 1980) with presaturation (γB_1_ = 50 Hz) during a relaxation delay of 4 s and a mixing time of 10 ms for efficient water suppression(Price 1999). Eight scans of 65,536 points covering 12,335 Hz were recorded and zero filled to 65,536 complex points prior to Fourier transformation, an exponential window function was applied with a line-broadening factor of 1.0 Hz. The spectra were manually phase and baseline corrected and automatically referenced to the internal standard (TSP = 0.0 ppm). Phase offset artifacts of the residual water resonance were manually corrected using a polynomial of degree 5 least square fit filtering of the free induction decay (Coron et al. 2001). In order to monitor proper filling of the NMR flow cell and for quality control 1D gradient profiles (Vanzijl et al. 1994) along the* z* axis were recorded for each sample prior and post data acquisition. Duration of 90° pulses were automatically calibrated for each individual sample using a homonuclear-gated nutation experiment (Wu and Otting 2005) on the locked and shimmed samples after automatic tuning and matching of the probe head.

### Statistical analysis

Each spectrum was integrated (binned) using 0.014 ppm integral regions between 10 and 1 ppm, the residual water and urea region between 6 and 4.5 ppm was excluded, resulting in 550 data points used for the analysis. To account for any difference in concentration between the samples, each spectrum was normalized to a total area of 1. Absolute values were log-transformed. All pre-processing was done using in-house developed routines in *R* statistical environment (http://www.r-project.org/). Variables were centered and unit variance scaled prior to statistical analysis in SIMCA-P+ (version 12.0; Umetrics, Sweden) software package. For initial analysis and outlier detection, principal component analysis (PCA) was performed using 10 components. After the initial PCA analysis the following regions corresponding to paracetamol and its metabolites were excluded from the analysis: 7.5–6.75, 3.95–3.8, 3.7–3.45, 2.2–2.14 and 1.84–1.88 ppm according to (Bales et al. 1985). For partial least squares-discriminant analysis (PLS-DA) (Nocairi et al. 2005) samples were categorized based on classes as defined by the study design. PLS model was built using 5 categories according to logarithm of bacterial count as a *Y* variable. Statistical models from supervised multivariate data analysis were validated by random permutation of the response variable and comparison of the goodness of fit (*R* ^2^ *Y* and *Q* ^2^) (Westerhuis et al. 2008; Lindgren et al. 1996). For random permutation tests 100 models were calculated and the goodness of fit was compared with the original model in a validation plot. Spectral regions responsible for the separation between classes in supervised models were identified based on the Variable Influence on Projection (VIP) values, which correspond to the importance of the variables (bins) for the model. The variables with a VIP value larger than 1.8 were considered significant and used for further analysis and identification of the responsible peak(s) within the spectrum. Prediction of class membership of samples by PLS-DA model was based on the predicted *Y* variable with the cut-off of 0.5.

For multilevel component analysis using an in-house developed script in *R* as described by Jansen et al*.* (2005). data were not log-transformed*.* To assess the predictive ability of multilevel PLS-DA in each cross-validation round all the samples, belonging to two random individuals were taken out.

Univariate tests were performed to assess the statistical significance of the spectroscopic regions found using multivariate analysis: unpaired t-test was performed for the regions found as discriminating between UTI patients and controls by PLS-DA; ANOVA was performed on the regions that showed association with bacterial count in PLS; paired *t* test was carried out on the regions identified in multilevel analysis. All the corresponding *P* values were adjusted for multiple testing using Benjamini–Hochberg correction.

### Identification of compounds of interest

Annotation of identified peaks was performed based on reference spectra from the Bruker Bioref database and in-house reference data. Confident identification was facilitated by the use of Statistical Total Correlation SpectroscopY method (Cloarec et al. 2005).

### Quantification of paracetamol and differential metabolites

Quantification was performed by deconvolution and subsequent integration of resonances, corresponding to the compounds of interest, using an in-house developed automation routine. Paracetamol–glucuronide was quantified based on the resonance at 5.10 ppm (*d*, 7.1 Hz); for other compounds the signal with high intensity and low degree of overlapping was chosen for quantification. The absolute concentrations were calculated based on internal reference TSP. Values were not corrected for differential attenuation of the signals caused by relaxation during the mixing time and rapid-pulsing saturation effects.

## Results

**Fig. 1:**
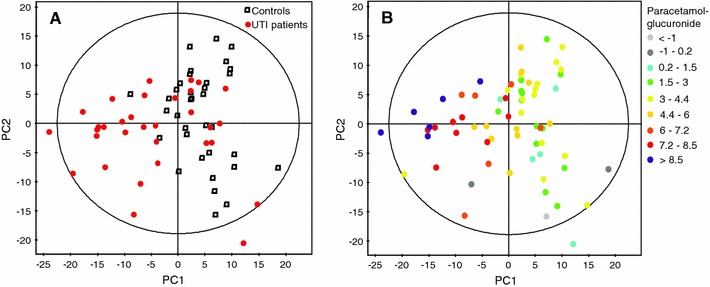
PCA scores plot of 1H NMR data from controls and UTI patients urine samples at baseline, first two principal components covering 14.5 and 10.2% of variation respectively. **a** *Colored* according to controls (*square*) and UTI patients (*circle*). **b** Colored according to the logarithm of absolute concentration of paracetamol–glucuronide (Color figure online)

The initial PCA on baseline samples revealed a trend in separation between UTI patients and controls in the scores plot of the first two principal components as shown in Fig. 1a. The loadings plot of this model was dominated by the spectral regions that belonged to one of the most commonly used over-the-counter analgesic, paracetamol (Supplementary Fig. 2). The absolute concentration of paracetamol-glucuronide was used to stratify samples in the PCA plot: the direction of increase of paracetamol-glucuronide was found to match the direction of controls-patients separation (Fig. 1b). As paracetamol is not an infection or morbidity marker, the further analysis was performed after the exclusion of the regions corresponding to the drug and its metabolites.

**Fig. 2:**
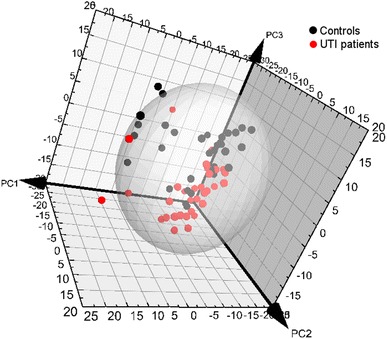
PCA scores plots of 1H NMR data from controls (*black*) and UTI patients (*red*) urine samples at baseline after removal of the regions corresponding to paracetamol and its metabolites. First principal component covers 11.7%, second 11.2% and third 9.8% of variation (Color figure online)

The PCA analysis of the baseline samples after the removal of spectral regions of paracetamol and its metabolites did not show separation between UTI patients and controls within the scores plot of the first two principal components; however, a clear trend was identified along the third principal component (Fig. 2), which means that inter-individual variability is to a certain extent more prominent than the disease effect. No outliers were detected based on distance to the model (DModX). The samples with other conditions than UTI (as defined in Table 2) did not exhibit any clustering in PCA scores plot.

**Table 2:** Spectroscopic regions that appear as influential in various statistical models and the statistical significance of the corresponding univariate tests based on the quantified corresponding peaks

Identity	ChemSpider IDs	Spectral bins (ppm)^a^	Controls vs. UTI patients^b^		Bacteria concentration^c^		Recovery from *t* = 0–30^d^	
			*t* test	Change	ANOVA *P* value	Change	Paired	Change
			*P* value				*t* test	
							*P* value	
1-Methylnicotinamide	10628510	9.284, 9.271, 8.971, 4.484	9.00E-07	↓	2.00E-05	↓		
Acetic acid	170	1.934, 1.921	7.00E-08	↑	2.00E-07	↑		
Acylcarnitine	NA	3.189	0.004	↑				
Citric acid	29081	2.562, 2.534	9.00E-05	↓				
Creatinine	568	4.075, 3.066, 3.052	0.3	↓				
Furoylglycine	20474156	7.703, 7.689					0.15	↑
Glycolic acid derivative	NA	3.953	0.55	↓	0.56	↓	0.55	↑
Hippuric acid	451	7.853, 7.662, 7.648, 7.580, 3.966, 8.548, 8.534	0.001	↓	0.001	↓		
Lactic acid	96860	1.334	0.0008	↑	0.0002	↑		
Para-aminohippuric acid	2063	7.757					0.055	↑
Scyllo-inositol	23975912	3.325					0.1	↑
Taurine	1091	3.448, 3.434, 3.421, 3.257	1.00E-03	↑	0.08	↑	2.00E-07	↓
Trigonelline	5369	8.698, 4.443					0.09	↑
Trimethylamine	1114	2.889	4.00E-06	↑	0.0001	↑	0.0001	
Unknown 1	NA	7.962					0.01	↑
Unknown 2	NA	7.743					0.015	↑
Unknown 3	NA	7.512			0.0006	↑		
Unknown 4	NA	6.68					4.00E-05	↑
Unknown 5	NA	6.503					0.065	↑
Unknown 6	NA	3.162			0.05	↓		

^a^Chemical shift corresponding to the center of the bin region

^b^Two-group *t* test for the healthy controls and UTI patients at baseline; ↑ corresponds to intensity of the region being higher in UTI patients compared to controls, ↓ means that region intensity is lower in UTI patients compared to controls

^c^ANOVA analysis for the number of bacteria present in urine; direction corresponds to the correlation to the number of bacteria: ↑ corresponds to the raise of the region intensity with the increase of the number of bacteria, ↓ to the decrease of the region intensity with the increase of the number of bacteria

^d^Paired *t* test for the UTI patients at baseline and 30 days; ↑ direction of change corresponds to intensity of the region being higher at 30 days compared to baseline, ↓ means that region intensity is lower at 30 days compared to baseline

**Fig. 3:**
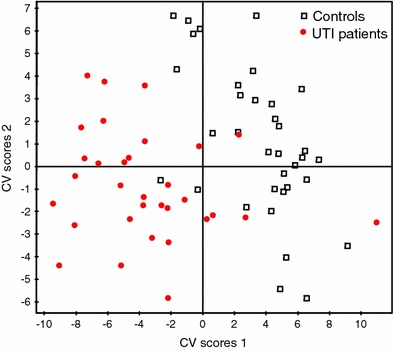
Cross-validated PLS-DA scores plot of urine 1H NMR spectra of controls (*square*) and UTI patients at baseline (*circle*), *R*2*Y* = 0.88, *Q*2 = 0.63

In the next step a supervised PLS-DA model was built for *t* = 0 using UTI/controls as a response variable. In the scores plot of the resulting model the two groups were well separated (Fig. 3). Cumulative explained variance (*R*2*Y*) of 0.88 and cross validated predictive fraction (*Q*2) of 0.63 were calculated for the model; the model validation plot showed intercepts of the *R*2*Y* and *Q*2 regression lines with the vertical axis at 0.63 and −0.11, respectively, indicating a valid model. Molecular discriminators were identified based on relevant regions as identified by the corresponding VIP. A list of those regions, along with the p-values based on t-test for the quantified compounds, the direction of change and identities of the corresponding metabolites are summarized in Table 2.

**Fig. 4:**
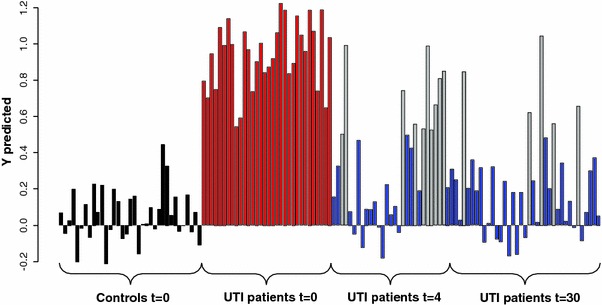
Predicted response value for two-class PLS-DA model based on controls (*black bars*) and UTI patients (*red bars*) at baseline: *blue bars* are the *t* = 4 and *t* = 30 classified as *controls*, *grey* are the *t* = 4 and *t* = 30 samples classified as UTI patients at *t* = 0 (Color figure online)

The advantage of PLS-based models is that they can easily be used to predict the class membership of new samples. Data of the UTI patients at *t* = 4 were predicted using the two-class PLS-DA model that was built as described above. Of a total of 29 urine samples included in the prediction set, 19 (65.5%) were classified as controls, whereas 10 (34.5%) samples were classified as UTI (Fig. 4). Besides using data from the 4-days time point as prediction set, we also performed a separate analysis for the 30-days time point (Fig. 4). In this case, out of 37 samples collected, 32 (86.5%) were attributed to the group of controls and 5 (13.5%) were categorized as UTI.

**Fig. 5:**
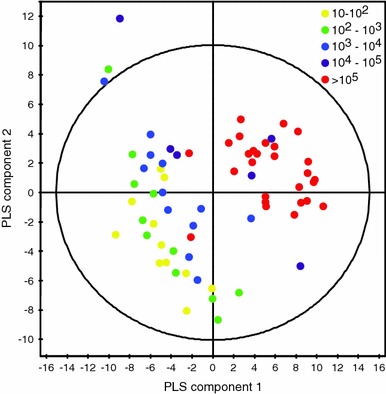
Scores plot of the PLS model of urine ^1^H NMR spectra at baseline versus the number of bacteria (CFU/ml) found in urine (*R*2*Y* = 0.78, *Q*2 = 0.44). *Colored* by the number of bacteria (Color figure online)

An important parameter characterizing UTI patients is the number of bacteria in urine; however, bacteria can also be present in urine of the individuals, who do not exhibit any symptoms of UTI (Lin and Fajardo 2008). We built a PLS regression model from NMR data of urine at baseline using the result of bacterial culture as response variable. Since bacterial count and UTI classification do not fully correlate we expected to obtain a slightly different model as compared to the model built based on UTI classification for this timepoint. Using 2 components a cumulative *R*2*Y* = 0.78 and *Q*2 = 0.44 were obtained and model validation showed intercepts of the *R*2*Y* and *Q*2*Y* regression lines with the vertical axis at 0.63 and −0.12, respectively, in the model validation plot. As can be seen from the PLS scores plot (Fig. 5) the samples with the highest bacteria concentration in urine were very distinct from the rest forming a separate cluster, whereas the rest of the samples were overlapping. The spectral regions responsible for the correlation of the ^1^H NMR data and bacterial count were chosen on the basis of the corresponding VIP. A list of those regions, along with the *P* values derived from ANOVA based on the quantified compounds, the direction of change and identities of the corresponding metabolites are summarized in Table 2.

To better understand the process of patient recovery and to find the spectroscopic regions that correlate with this process, we took advantage of the longitudinal study design. One of the statistical methods suitable for such analysis is multilevel component analysis that separates variation present in the data into two levels: between-individual and within-individual. We performed this analysis on the 29 patients for which both the data from the baseline and from the 30-days time point were available and concentrated on the within-individual information. This should best reflect the recovery from the baseline, when patients are diagnosed as infected, to 30 days, when they are considered UTI symptom-free. PCA scores plot of the first two principle components that cover 15.8 and 14.8% of the variation, respectively, showed good separation between baseline and *t* = 30 time points (data not shown). The PLS-DA model of this data had high quality parameters (*R*2*Y* = 0.98, *Q*2 = 0.78 for four components), performs significantly better then random models (*P* < 10^−15^) and perfectly separated the two time points (data not shown). The NMR spectral regions responsible for the separation between baseline and the t = 30 time point were identified based on VIP values. The underlying metabolites as well as the p-values from paired t-test based on the quantified compounds and the direction of change are summarized in Table 2.

To summarize, taking advantage of our study design we built several statistical models, which allowed getting a broader view of metabolic features associated with UTI, than the traditional “case–control” design.

## Discussion

UTI, as many other disorders, represents a complex clinical entity, for which diagnostics is not straightforward and based on consensus criteria (Wilson and Gaido 2004). Thus, unlike in animal experiments, in clinical research assigning people to certain groups is not always unconditional. The diagnosis of a disease can be ambiguous and defining the healthy group can be complicated by that there is no clear definition of “healthy”. Consequently, it may be very advantageous to supplement a traditional “case–control” design with a more complex study design and the use of additional clinical data. When used without extra information, “case–control” analysis might even be misleading. For example, the separation of the control and UTI groups was seen in the first two principal components of PCA; however, this discrimination was not disease-related, but the result of patients taking the antipyretic and analgesic drug paracetamol. An analysis strategy for such type of data is to identify all of the spectroscopic regions that contain signals from drug-related compounds and to exclude them prior to further analysis. However, it is not feasible to account for the whole range of the medication used and, more importantly within the context of clinical metabolomics studies in general, to account for drug-related shifts in metabolism, especially in the case of long-term treatment regimes of chronic conditions. It is essential to consider such effects when developing the study design in order to minimize or control such influences.

Samples from 4 days after admission, when the patients were still under therapy, but on the way to recovery, were used to check if the modeled differences were related to the effect of medication or not. The fact that the majority of those samples were classified as healthy by the model built on baseline samples is an indication that the model is not reflecting therapy/drug intake, but is indeed related to the clinical difference between the groups.

The samples from the 30-days time point, when UTI patients were symptom-free, could also be used to gain additional information on the performance of the model as well as to get insight into the underlying biology. When predicted using the PLS-DA model built on the baseline UTI infected and UTI symptom-free samples, most of the 30-days samples (86.5%) were projected to the control group. Those few, which were still predicted as infected UTI patients, may have another condition (as we do not know at this point how specific our model is) or have asymptomatic UTI. On the other hand, they can be healthy and be false positives, as the predictive ability of our model, estimated by cross-validation was 63%. There was neither a consistent pattern of metabolites that would describe the prediction of 30-days samples as infected, nor common complication/co-morbidity between those samples, which makes it difficult to suppose the reason for the misprediction. Despite that, considering the prediction of 30-days samples as an independent statistical test for our model, it gives very satisfactory results.

Pair-wise analysis for baseline and 30-days samples from the same individuals was conducted in order to monitor the recovery process. It revealed a number of classifiers and improved their statistical significance. The identified metabolites overlapped with the compounds from the model discriminating healthy and UTI subjects, however a few of them were unique (para-aminohippuric acid, scyllo-inositol and a few unidentified compounds).

Besides the multilevel design, the advantage of the current study was the exhaustive clinical characterization of the patients. Among the variety of clinical parameters available, the number of bacteria in urine was of specific importance. We performed regression-based analysis of the relation between the ^1^H NMR data and the bacterial load in urine as determined by bacterial culture. The classifiers that emerged from this analysis were to a certain extent overlapping with the classifiers derived from the discriminative model on baseline samples. This was no surprise, since UTI is generally characterized by the presence of bacteria in urine.

Straightforward assignment of classifiers from each model to the intracellular biochemical pathways will not improve the understanding of UTI. We tried to use the information available from the different statistical models jointly in order to enrich the biological interpretation. When comparing the lists of discriminators obtained from the different models (discriminating UTI patients from controls, modeling the recovery process and modeling the data against the degree of bacterial contamination of urine) it is evident that there is a large overlap which makes biological interpretation of the results feasible. For instance, some of the overlapping metabolites were already known from the literature to be related to the bacterial contamination of urine: acetate, lactate and trimethylamine (Gupta et al. 2009). Others, if they were found only in the comparative analysis of the two groups, could be attributed based on previous studies to certain phenomena. Hippuric acid, for example, is often associated with the gut microflora (Swann et al. 2009) and taurine with liver toxicity (Nicholson et al. 1999). However, our findings suggest that they are also associated with the bacterial contamination of urine, which obviously does not mean that they are not related to the mentioned physiological processes as well, but that a complex network of interconnected factors is involved. The metabolites that appear to be related to the recovery process might be considered as potential morbidity markers. One of them, para-aminohippuric acid, is a well-established diagnostic marker for renal plasma flow and glomerular filtration (Reubi 1953). The recovery from the complicated, tissue-invasive UTI is associated with the resumption of the kidneys’ function, so the positive change in para-aminohippuric acid corroborates our assumption that some of the markers discovered in the paired analysis are the markers of morbidity.

Despite the promising discriminatory and predictive abilities of the statistical models, described above, their real clinical utility would need careful examination. As a first attempt we restricted ourselves to the case of *E. coli* as the main pathogen of UTI. Currently we are planning to investigate if our models will hold when samples with other pathogens are added. Besides that, the specificities of the models with regard to other diseases of both infectious and non-infectious origin have to be assessed.

## Conclusions

In the current paper we used a metabolomics approach to profile UTI, which is on the one hand one of the most common infectious diseases among the adults, and on the other hand a disease that still lacks markers of morbidity. Using ^1^H NMR profiles of urine we generated various statistical models: (a) discriminating UTI patients and control subjects, (b) following the recovery process of UTI patients and (c) associating urine metabolic content with bacterial contamination. The discriminative model was able to classify most of the independent samples correctly according to their diagnosis, which indicates its high predictive ability. Comparing the sets of molecules derived from different analyses, we concluded that some of the compounds (e.g. trimethylamine and acetate) can be attributed to the effect of bacterial contamination of urine; others (e.g. para-aminohippuric acid, scyllo-inositol) can be considered markers of morbidity.

## Electronic supplementary material

Below is the link to the electronic supplementary material.

Supplementary material 1 (DOC 720 kb)

Supplementary material 2 (XLS 774 kb)

Supplementary material 3 (XLS 24 kb)
