# Supplementary material for: 1H NMR-based metabolic profiling of urinary tract infection: combining multiple statistical models and clinical data
Source: Metabolomics. 2012 Feb 29;8(6):1227–35. doi: 10.1007/s11306-012-0411-y (PMC3483096; doi:10.1007/s11306-012-0411-y)
Supplement: Supplementary file 1 — Supplementary material 1 (DOC 720 kb) [file 11306_2012_411_MOESM1_ESM.doc]

**Figure 1. Flowchart of participants in the study (A) and design of the NMR study.**

**Figure 2. Loadings plot of the PCA model created using urine spectra of samples at baseline. Dots indicate variables that correspond to the spectral regions of paracetamol and its metabolites, triangles represent all the other variables.**

The original table with binned spectra (created as described in the Materials and Methods) is in the supplementary comma-separated file **bucket_table.csv**. The supplementary file **Meta_data.xls** contains the corresponding table with additional data used for the analysis (degree of bacterial infection, timepoint, etc.).
